# Pandemiebedingte Belastungserfahrungen, Ressourcen und depressive Stimmungen von Studierenden am Ende des Online-Wintersemesters 2020/21

**DOI:** 10.1007/s11553-022-00949-x

**Published:** 2022-05-09

**Authors:** Carina Förster, Anja Hawlitschek, Rahim Hajji

**Affiliations:** Fachbereich Soziale Arbeit, Gesundheit und Medien, Hochschule Magdeburg-Stendal, Breitscheidstraße 2, 39114 Magdeburg, Deutschland

**Keywords:** Psychisches Wohlbefinden, Belastungen, Coping, Soziale Unterstützung, Resilienz, Mental wellbeing, Stress, Coping, Social support, Resilience

## Abstract

**Hintergrund:**

Studierende zählen weltweit zu einer vulnerablen Gruppe, welche überdurchschnittlich zu depressiven Störungen neigt. Empirische Untersuchungen zeigen auch, dass depressive Stimmungen unter Studierenden deutlich zugenommen haben, während der COVID-19-Pandemie („coronavirus disease 2019“).

**Fragestellung:**

Das Ziel des Artikels ist zu untersuchen, ob die pandemiebedingten Belastungserfahrungen im Zusammenhang mit der depressiven Stimmung der Studierenden stehen. Darüber hinaus wird analysiert, ob Resilienz, Coping und soziale Unterstützung als Ressourcen mit den depressiven Stimmungen von Studierenden assoziiert sind. Dabei soll geklärt werden, welche Ressourcen den Zusammenhang zwischen den pandemiebedingten Belastungserfahrungen und der depressiven Stimmung von Studierenden als Mediatoren erklären können.

**Methodik:**

Die Studie beruht auf einer Online-Befragung von Studierenden der Hochschule Magdeburg-Stendal zum Wintersemester 2020/2021. Insgesamt sind die Daten von 621 Studierenden ausgewertet worden. Zur Beantwortung der Fragestellung wurde eine Mediationsanalyse durchgeführt.

**Ergebnisse:**

Die pandemiebedingten Belastungserfahrungen stehen in einem signifikanten Zusammenhang mit den depressiven Stimmungen von Studierenden. Die Resilienz stellt ebenfalls einen signifikanten Einflussfaktor zu den depressiven Stimmungen dar und mediiert partiell den Einfluss von den pandemiebedingten Belastungserfahrungen auf die depressive Stimmung von den Studierenden. Coping und soziale Unterstützung weisen keinen signifikanten Zusammenhang zu der depressiven Stimmung der Studierenden auf.

**Schlussfolgerungen:**

Ansatzpunkte zur Reduzierung der depressiven Stimmung liegen in der Minderung der pandemiebedingten Belastungen und in der Stärkung der Resilienz der Studierenden.

## Abstract (en)

**Background:**

Students worldwide belong to a vulnerable group with an above-average tendency towards depressive disorders. Empirical studies also show that depressive moods among students have increased significantly during the coronavirus disease 2019 (COVID-19) pandemic.

**Objectives:**

The aim of the article is to examine whether the stress experiences caused by the pandemic are related to the depressed mood of the students. In addition, it is analyzed whether resilience, coping and social support as resources are associated with the depressive moods of students. The aim is to clarify which resources can explain the connection between the stress experiences caused by the pandemic and the depressive mood of students as mediators.

**Methods:**

The study is based on an online survey of students at Magdeburg-Stendal University of Applied Sciences for the 2020/21 winter semester. The data of 621 students were evaluated. A mediation analysis was carried out.

**Results:**

Pandemic-related stress experiences are significantly related to the depressive moods of students. Resilience is also a significant factor influencing depressive moods and partially mediates the influence of pandemic-related stressful experiences on the depressed mood of the students. Coping and social support show no significant association with the depressed mood of the students.

**Conclusion:**

Starting points for reducing the depressive mood lie in reducing the stress caused by the pandemic and in strengthening the resilience of the students.

## Hintergrund und Fragestellung

Studierende zählen weltweit zu einer vulnerablen Gruppe, die überdurchschnittlich zu psychischen Problemen und depressiven Störungen neigt [3, 16, 18]. Empirische Studien zeigen, dass die Prävalenz von Depression unter Studierenden während der Pandemie von 19,7 % auf 31,2 % gestiegen ist [3, 8]. Auch durchgeführte Trendstudien an der Hochschule Magdeburg-Stendal belegen, dass die depressiven Stimmungen unter Studierenden von 11 % auf 25 % zugenommen haben [13]. Die depressive Stimmung wird als das Wahrnehmen von Symptomen der Depression, wie beispielsweise das Gefühl von Traurigkeit, emotionaler Niedergeschlagenheit, Antriebslosigkeit oder Unlust, verstanden [10, S. 15–17].

Zur Erklärung der depressiven Stimmung kann das Vulnerabilitäts-Stress-Modell herangezogen werden [21]. Zu den Ursachen einer Depression können nach Brakemeier et al. [5] (neuro)biologische, psychologische und umweltbezogene Vulnerabilitätsfaktoren gezählt werden, die die individuelle Anfälligkeit für psychische Störungen ausmachen. In dem Vulnerabilitäts-Stress-Modell sind die Merkmale der psychischen Störung das Ergebnis des Zusammenwirkens der Vulnerabilität einer Person mit den erlebten Stressereignissen.

Zu den Stressereignissen können nach Wittchen und Hoyer [21] kritische Lebensereignisse gezählt werden. In der vorliegenden Untersuchung wird die COVID-19-Pandemie („coronavirus disease 2019“) und die damit verbundenen Maßnahmen zur Eindämmung der Krankheit als coronabedingte Stressoren betrachtet. Die notwendige pandemiebedingte Schließung von Hochschulen und Universitäten im Sommersemester 2020, Wintersemester 2020/2021 und im Sommersemester 2021 hat dazu geführt, dass die Lebenswelt der Studierenden sich veränderte. Die Umstellung von Präsenz- auf Online-Lehre und die damit einhergehende Änderung von Lehr-Lern-Prozessen und Lehr-Lern-Verhalten werden von den Studierenden als gravierende Belastungen eingeschätzt [11]. Insbesondere reduzierte Interaktionen mit anderen Studierenden und den Lehrenden werden von den Studierenden als negativ beschrieben [2, 15]. Aber auch die gestiegenen Anforderungen an die Selbstlernkompetenzen werden als Belastung empfunden. Die Studierenden berichten über Probleme bei der Konzentration, Motivation und Selbstorganisation [1, 2] und über Unsicherheiten oder Misserfolgsängste [12]. Hinzu kommt ein subjektiv wahrgenommener erhöhter zeitlicher Aufwand für das Studium aufgrund der Umstellung auf Online-Lehre [2]. Empirische Studien zeigen u. a., dass die pandemiebedingten Belastungen im Studium in einem signifikanten Zusammenhang mit psychischen Problemen [22] stehen.

In Anknüpfung an das Vulnerabilitäts-Stress-Modell können psychologische Faktoren wie Coping [18], Resilienz [7] und soziale Unterstützung [23] als potenzielle Ressourcen die psychischen Störungen mit ihrer entsprechenden Symptomatik unterhalb der Erkrankungsschwelle halten [20, S. 250].

Die Autorengruppe um Ye et al. [22] hat den Einfluss vom Stresserleben durch die COVID-19-Pandemie auf eine akute Belastungsstörung unter Berücksichtigung eines mediierenden Effekts durch Resilienz, Coping und soziale Unterstützung bei Studierenden untersucht. Die Studie zeigt, dass die Ressourcen, den Einfluss von Stresserleben auf die psychische Störung mindern (*p* < 0,001). Das pandemiebedingte Stresserleben beeinflusst die subjektive Verfügbarkeit der genannten Ressourcen negativ, während eine höhere Ressourcenverfügbarkeit mit einer geringeren Ausprägung der psychischen Störung in Verbindung gebracht wird. Ye et al. [22] weisen auch nach, dass Resilienz, Coping und soziale Unterstützung als Mediatoren zwischen pandemiebedingten Stresserleben und psychischen Störungen wirksam sind. Das heißt, dass starke pandemiebedingte Belastungserfahrungen in einem Zusammenhang mit geringen Ressourcen und die Verfügbarkeit von geringen Ressourcen in einem Zusammenhang mit psychologischen Problemen stehen.

Das Ziel des vorliegenden Beitrags ist es (1) zu untersuchen, inwieweit die wahrgenommene pandemiebedingte Belastung der Studierenden im Studium im Zusammenhang mit depressiven Stimmungen steht und (2) welche Ressourcen (Coping, Resilienz, soziale Unterstützung) den Zusammenhang zwischen der Belastung und der depressiven Stimmung von Studierenden vermitteln bzw. mediieren. Diese Fragestellung ermöglicht es zu untersuchen, wie stark die Folgen der pandemiebedingten Belastungserfahrungen auf die depressiven Stimmungen der Studierenden sind und welche Ressourcen der Studierenden den Zusammenhang zwischen der pandemiebedingten Belastungserfahrung und der depressive Stimmung aufklären können. Auf diese Weise können soziale Mechanismen identifiziert werden und Ansatzpunkte für die Gesundheitsförderung und Prävention entwickelt werden.

**Abb. 1:**
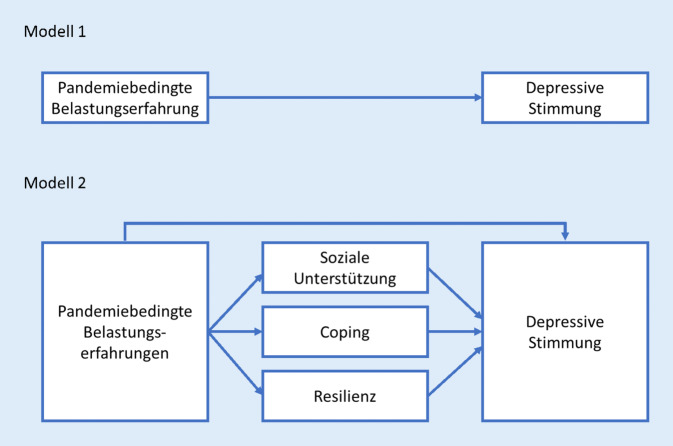
Modellierung der Fragestellung

Zusammenfassend lässt sich die Fragestellungen anhand der folgenden Modelle visualisieren (Abb. 1):

## Methodische Herangehensweise

### Studiendesign

Die Analysen der vorliegenden Arbeit beruhen auf einer Online-Befragung von Studierenden der Hochschule Magdeburg-Stendal zum Ende des Wintersemesters 2020/21. 621 Studierende nahmen in einem Zeitraum vom 29. März 2021 bis zum 7. April 2021 an der Online-Befragung teil. Dies entspricht einer Rücklaufquote von 11 %.

### Operationalisierung

Die Operationalisierung der Theoriebegriffe lässt sich anhand der Tab. 1 ablesen. Die Messung der depressiven Stimmung bezog sich auf das Erleben von depressiven Symptomen. Die Erhebung der sozialen Unterstützung beruhte auf der Einschätzung, wie oft man Unterstützung von Kommilitonen erhalten hat. Die Frage zum Coping nimmt Bezug auf die erfolgreiche Bewältigung von herausfordernden Situationen und die Frage zur Resilienz bezieht sich auf die Fähigkeit sich schnell bei Belastungen erholen zu können.

**Tab. 1:** Operationalisierung

Theoriebegriff	Indikator	Antwortkategorien
Depressive Stimmung	Wie häufig fühlen Sie sich depressiv?	(1) nie bis (5) immer
Pandemiebedingte Belastungserfahrungen (Mittelwertindex gebildet); Cronbach’s Alpha = 0,8	Wie sehr belastete die Coronapandemie im Wintersemester 2020/2021?	(1) belastete mich gar nicht bis (5) belastete mich sehr
	… Ihr persönliches Lernverhalten	
	… Ihre Konzentrationsfähigkeit beim Lernen	
	… Ihre Lernmotivation	
	… Ihre Noten im Studium	
	… Das gemeinsame Lernen mit anderen	
	… Ihren inhaltlichen Austausch mit Lehrenden	
	… Ihre persönlichen Lebensbedingungen	
Soziale Unterstützung durch Studierende	Wie häufig erhalten Sie bei Bedarf Hilfe von Ihren Kommilitonen?	(1) nie bis (5) immer
Coping	Wie häufig gelingt es Ihnen herausfordernde Situationen erfolgreich zubewältigen?	(1) nie bis (5) immer
Resilienz	Wie häufig gelingt es Ihnen sich bei hohen Belastungen schnell zu erholen?	(1) nie bis (5) immer
*Kontrollvariablen*		
Geschlecht	Sind Sie	Männlich, weiblich, divers?
Alter	Wie alt sind Sie?	–
Nachteilsausgleich im Studium	Sind Sie Besitzer/in eines KomPasses?	Ja; nein; weiß nicht, was das ist
Wohnform	Wo haben Sie während des Wintersemesters 2020/2021 gelebt?	In einer eignen Wohnung; in einer WG; bei den Eltern; andere Wohnform
Aufwand für das Studium	Wenn Sie an das Wintersemester 2020/2021 denken, wie würden Sie Ihren Aufwand für das Studium insgesamt beurteilen?	(1) sehr gering bis (5) sehr hoch
Prüfungsangst	Wie häufig haben Sie in den Prüfungen im Wintersemester 2020/2021 an die Konsequenzen eines Scheiterns gedacht?	(1) nie bis (5) immer

Die Erhebung der genannten Theoriebegriffe mit Single-Items lässt diesbezüglich eine geringere Reliabilität und Validität vermuten. Für die Erhebung der depressiven Stimmung liegen jedoch Hinweise vor, dass kurze Messinstrumente, wie das verwendete Single-Item, gleich gute Ergebnisse liefern können, wie lange Messinstrumente (vgl. [17]).

Die pandemiebedingten Belastungserfahrungen wurden mittels sieben Aussagen (u. a. „Wie sehr belastete die Coronapandemie im Wintersemester 2020/2021 ihr persönliches Lernverhalten?“) erhoben. Die Reliabilität ist mittels dem Cronbachʼs Alpha berechnet worden und liegt mit 0,8 in einem akzeptablen Bereich, so dass die Messung der pandemiebedingten Belastungserfahrungen mittels der Bildung einer Mittelwertskala vorgenommen worden ist.

Das Geschlecht, das Alter, der Nachteilsausgleich im Studium, die Wohnform, die Einschätzung des Aufwands für das Studium und die Prüfungsangst wurden als Kontrollvariablen berücksichtigt.

### Stichprobenbeschreibung

Die Tab. 2 zeigt die soziodemografischen Angaben der Stichprobe. 61 % der befragten Studierenden sind weiblich, 38 % männlich und 0 % divers. Mit einem Anteil von 62 % ist der Großteil der Stichprobe 23 Jahre und jünger. 55 % der Studierenden wohnt in einer eigenen Wohnung. Lediglich 7 % der Stichprobe besitzt einen Pass zur Kompensation besonderer Aufgaben (KomPass). Der KomPass ist ein Instrument zum Nachteilsausgleich der Hochschule Magdeburg-Stendal und kann von Studierenden mit Erkrankungen, Handicaps und Sorgeaufgaben beantragt werden.

**Tab. 2:** Stichprobenbeschreibung

	*n*	%
*Rücklaufquote*	621	11
*Geschlecht*		
Männlich	233	38
Weiblich	381	61
Divers	3	0
*Alter (Jahre)*		
Unter 21	155	25
21–23	227	37
24–26	122	20
27–29	40	6
30 und mehr	76	12
*Wohnform*		
In eigenen Wohnung	342	55
In einer Wohngemeinschaft (WG)	119	19
Bei den Eltern	141	23
In einer anderen Wohnform	16	3
*KomPass-Inhaber*		
Ja	41	7
Nein	332	53
Ich weiß nicht, was das ist	245	39

### Auswertungsstrategie

Zur empirischen Untersuchung der Fragestellung wurden uni-, bi- und multivariate Analysen mit SPSS von IBM und Process [14] durchgeführt. Die multivariate Analyse erfolgte anhand einer Mediationsanalyse. Diese beruht im ersten Schritt (Modell 1) darauf zu untersuchen, ob die pandemiebedingte Belastungserfahrung in einem signifikanten Zusammenhang mit der depressiven Stimmung steht. Im zweiten Schritt (Modell 2) wird der Frage nachgegangen, welche Ressourcen den Zusammenhang zwischen pandemiebedingten Belastungserfahrungen und depressiver Stimmung als Mediator aufklären kann. Die empirische Untersuchung berücksichtigt dabei auch Faktoren, wie Geschlecht, Alter, gesundheitliche Beeinträchtigungen, die Wohnform, der Aufwand für das Studium und die Prüfungsangst.

## Ergebnisse

### Univariate Ergebnisse

Die Tab. 3 weist die deskriptiven Ergebnisse aus.

**Tab. 3:** Deskriptive Ergebnisse

	*n*	%
*Gesundheitsbezogene Indikatoren*		
Depressive Stimmung (oft, immer)	157	25
Pandemiebedingte Belastungserfahrungen (belastete mich mittelmäßig bis sehr)	367	59
*Studienbedingungen*		
Aufwand für das Studium (hoch, sehr hoch)	359	58
Prüfungsangst (oft, immer)	212	34
*Ressourcen*		
Soziale Unterstützung (oft, immer)	361	58
Coping (oft, immer)	411	66
Resilienz (oft, immer)	225	36

Mit 25 % fühlt sich etwa jeder vierte Studierende oft oder immer depressiv. Die pandemiebedingten Belastungserfahrungen werden von 59 % der Studierenden als mittelmäßig bis sehr belastend eingeschätzt. Der Studienaufwand wird von 58 % der Studierenden als hoch bis sehr hoch bezeichnet. 34 % der Studierenden haben in der Prüfungssituation oft oder immer Angst vor dem Scheitern erlebt. 58 % der Studierenden kann sich oft bis immer auf die Unterstützung von Kommiliton:innen verlassen und 66 % der Studierenden kann oft bis immer herausfordernde Situationen erfolgreich bewältigen. Eine schnelle Erholung nach hohen Belastungen kann lediglich 36 % der Studierenden oft bzw. immer bei sich wahrnehmen.

### Bivariate Ergebnisse

Die Ergebnisse der Korrelationsanalyse sind in Tab. 4 dargestellt. Die pandemiebedingten Belastungserfahrungen stehen erwartungsgemäß im signifikanten Zusammenhang mit den depressiven Stimmungen (*p* < 0,01). Das heißt, Studierende, die sich durch die Pandemie belastet fühlen, weisen eher depressive Stimmungen auf. Auch die Prüfungsangst als ein Indikator der Studienbedingungen steht in einem signifikanten positiven Zusammenhang (*p* < 0,01) mit der depressiven Stimmung. Studierende, die tendenziell zu Prüfungsangst neigen, tendieren eher zu depressiven Stimmungen. Der Studienaufwand steht in keinem Zusammenhang mit der depressiven Stimmung.

**Tab. 4:** Korrelationsanalyse nach Pearson

Korrelationsanalyse nach Pearson		A	B	C	D	E	F	G
*Gesundheitsbezogene Indikatoren*								
A	Depressive Stimmung	1	–	–	–	–	–	–
B	Pandemiebedingte Belastungserfahrungen	0,53^a^	1	–	–	–	–	–
*Studienbedingungen*								
C	Aufwand für das Studium	0,05	0,12^a^	1	–	–	–	–
D	Prüfungsangst	0,43^a^	0,48^a^	0,15^a^	1	–	–	–
*Ressourcen*								
E	Soziale Unterstützung durch Studierende	−0,18^a^	−0,20^a^	−0,05	−0,17^a^	1	–	–
F	Coping	−0,43^a^	−0,49^a^	0,01	−0,40^a^	0,22^a^	1	–
G	Resilienz	−0,53^a^	−0,52^a^	−0,22^a^	−0,37^a^	0,20^a^	0,44^a^	1

^a^Die Korrelation ist auf dem Niveau von 0,01 (2-seitig) signifikant

Die erhobenen Ressourcen stehen in einem signifikanten, negativen Verhältnis (*p* < 0,01) mit der depressiven Stimmung. Demnach weisen Studierende, die soziale Unterstützung durch Studierende erleben, erfolgreich herausfordernde Situationen bewältigen können und sich schnell von belastenden Situationen erholen können, eher eine geringere Neigung zu depressiven Stimmungen auf.

### Multivariate Ergebnisse

**Abb. 2:**
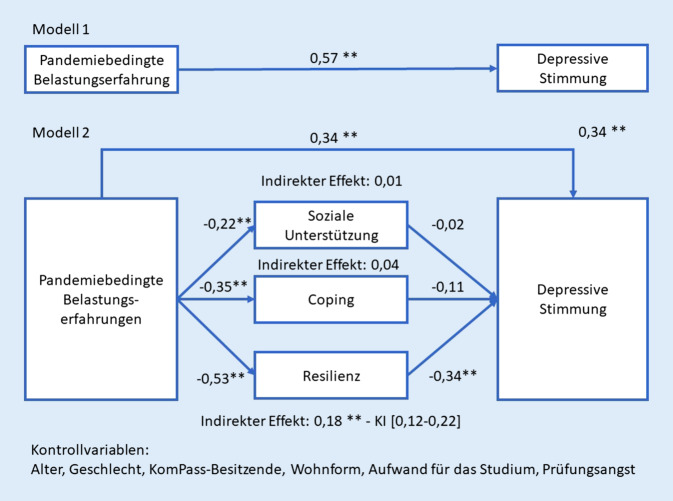
Mediationsmodell nach Hayes [14] zur Erklärung der depressiven Stimmung ( **Die Korrelation ist auf dem Niveau von 0,01 [2-seitig] signifikant)

Eine Mediationsanalyse (Abb. 2) ist mit dem von Andrew F. Hayes entwickelten SPSS-Modul „Process“ gerechnet worden [14]. Im Modell 1 ist der direkte Effekt der pandemiebedingten Belastungen auf die depressive Stimmung von Studierenden mit einem Koeffizienten von 0,57 (*p* < 0,01) signifikant. Demnach kann für die erste Fragestellung festgestellt werden, dass die pandemiebedingten Belastungen, ohne dabei die Ressourcen der Studierenden zu berücksichtigen, in einem signifikanten Zusammenhang mit der depressiven Stimmung der Studierenden steht.

Im Modell 2 verringert sich der direkte Zusammenhang der pandemiebedingten Belastungserfahrungen unter Berücksichtigung der Ressourcen auf die depressive Stimmung um 40 % (= 0,34/0,57), verliert jedoch nicht an Signifikanz. Das heißt, dass unter Berücksichtigung der Ressourcen der Studierenden, die pandemiebedingte Belastungserfahrungen weiterhin in einem signifikanten Verhältnis zu der depressiven Stimmung ist, aber nicht mehr so stark wie im Modell 1.

Des Weiteren ist im Modell 2 ablesbar, dass die pandemiebedingten Belastungen in einem signifikanten negativen Zusammenhang (*p* < 0,01) mit den Ressourcen stehen. Das heißt, je höher die pandemiebedingten Belastungen wahrgenommen werden, desto geringer werden die eigenen Ressourcen, wie die soziale Unterstützung durch Studierende, die eigene Coping-Fähigkeit und die individuelle Resilienz wahrgenommen.

Die einzelnen Ressourcen in dem Mediationsmodell vermitteln den Effekt von der pandemiebedingten Belastungserfahrungen auf die depressiven Stimmungen der Studierenden unterschiedlich stark. Während die Resilienz einen eindeutigen signifikanten mediierenden Effekt in dem Mediationsmodell ausweist (indirekter Effekt: 0,18; *p* < 0,01), ist dies bei Coping und sozialer Unterstützung nicht festzustellen.

Aufgrund der Analyse lässt sich feststellen, dass wenn die pandemiebedingte Belastungserfahrung um eine Einheit steigt, sich als direkter Effekt die depressive Stimmung um 0,34 Einheiten erhöht. Der indirekte Effekt lässt sich mit folgendem Beispiel veranschaulichen: Wenn die pandemiebedingte Belastungserfahrung um eine Einheit steigt, dann mindert sich die Resilienz um 0,53 Einheiten und dies hat die Folge, dass die depressive Stimmung um 0,18 Einheiten steigt.

## Diskussion

### Zusammenfassung der Ergebnisse

Die vorliegende Untersuchung bestätigt anhand einer Mediationsanalyse demnach, dass die pandemiebedingten Belastungserfahrungen in einem signifikanten Zusammenhang mit der depressiven Stimmung der Studierenden steht (Frage 1) und auch indirekt über die Resilienz mit der depressiven Stimmung von Studierenden assoziiert ist. Es handelt sich bei der Berücksichtigung der Resilienz als Mediator zur Erklärung des Zusammenhangs zwischen den pandemiebedingten Belastungserfahrungen und der depressiven Stimmung, nicht um eine vollständige, sondern um eine partielle Mediation (Frage 2). Ein signifikanter Zusammenhang von Coping und sozialer Unterstützung auf die depressive Stimmung kann mit der vorliegenden Untersuchung nicht beobachtet werden.

### Interpretation

Das Vulnerabilitäts-Stress-Modell verweist darauf, dass psychische Störungen u. a. das Ergebnis aus dem Zusammenwirken von Stressereignissen und psychologischen Faktoren (wie die Ressourcen) sind. Die sozial-psychologischen Mechanismen sind weitestgehend noch unerforscht [21].

Die vorliegende Analyse zeigt, dass die pandemiebedingten Belastungserfahrungen in einem negativen Zusammenhang mit den Ressourcen stehen. Es ist anzunehmen, dass die COVID-19-Pandemie und die notwendigen Coronamaßnahmen im Wintersemester 2020/2021 zur Eindämmung der Pandemie eine besondere Herausforderung für Studierende darstellt. Die Schließung von Hochschulen, Bibliotheken, Cafés etc., die Umstellung auf Online-Lehre sowie die herrschenden Kontakteinschränkungen dürften für Studierende eine Belastung darstellen, die sich niederschlägt in der Äußerung einer niedrigen Ressourcenausstattung. Es bleibt dabei unklar, ob die Ressourcen durch die Belastungserfahrung aufgebraucht werden oder ob der Ressourcenbedarf mit der Belastungserfahrung zugenommen hat oder ob die Belastungserfahrung dazu führt, dass man die Ressourcenausstattung schlechter einschätzt, obwohl diese nach wie vor vorhanden ist. Es ist anzunehmen, dass die angeführten Gründe in verschiedenem Ausmaß den Zusammenhang zwischen der pandemiebedingten Belastungserfahrung und den Ressourcen erklären dürften.

Die pandemiebedingten Belastungserfahrungen stehen auch unter Berücksichtigung von Ressourcen in einem signifikanten Zusammenhang mit den depressiven Stimmungen. Der direkte Effekt der pandemiebedingten Belastungserfahrungen lässt sich mit den Ressourcen nicht aufklären. Die Erfahrung von pandemiebedingten Belastungen steht demnach mit der subjektiven Erfahrung von depressiver Stimmung in Verbindung. Darüber hinaus wirkt die pandemiebedingte Belastungserfahrung auf die Resilienz mindernd und wirkt damit indirekt erhöhend auf die depressiven Stimmungen der Studierenden.

Zur Förderung der psychischen Gesundheit von Studierenden ergeben sich daraus zwei Ansatzpunkte. Zum einen sollten die pandemiebedingten Belastungserfahrungen reduziert werden, so dass der direkte und indirekte Effekt der pandemiebedingten Belastungserfahrungen auf die depressiven Stimmungen gemindert werden kann. Zum anderen gilt es die Resilienz der Studierenden zu stärken, damit die Regenerationsfähigkeit der Studierenden gefördert wird.

Zur Minderung der pandemiebedingten Belastungserfahrungen könnte eine sorgfältige didaktische Planung – die in der Ad-hoc-Umstellungsphase im Wintersemester 2020/2021 kaum möglich war – beitragen. Bereits in der Planungsphase für Online-Lernaktivitäten sollten Lehrende überlegen, wie Interaktionen zwischen Lernenden und Lernenden sowie zwischen Lernenden und Lehrenden angeregt werden können und Belastungserfahrungen abgebaut werden. Als sinnvoll wahrgenommene Interaktionen führen zu steigender Persistenz, Zufriedenheit und Lernerfolg beim Online-Lernen [6].

Zur Förderung der Resilienz der Studierenden existieren eine Reihe von evidenzbasierten Interventionsmöglichkeiten (vgl. [9]). Erwähnt sei beispielsweise Maßnahmen zur Förderung der Selbstfürsorge, Achtsamkeit und Study-Life-Balance, die darauf abzielen über die Selbstregulation die Resilienz zu stärken. Die Ergebnisse der Studie von Hahn et al. [11] deuten darauf hin, dass ein besonders großes Belastungserleben im Studium während der Coronapandemie mit einer niedrigeren Selbstlern- bzw. Selbstorganisationskompetenz einhergeht, die als fehlende Fähigkeit zur Selbstregulation verstanden werden kann. Dies könnte ein Ansatzpunkt für Interventionen sein. Online-Lehre erfordert ein hohes Maß an Selbstlern- und Selbstorganisationsfähigkeiten der Lernenden (vgl. [19]). Studierende, bei denen diese Kompetenzen nicht im ausreichenden Maße vorhanden sind, benötigen Unterstützung. Wenn die Selbstregulation während des Studiums kontinuierlich gefördert wird, erwachsen daraus jedoch nicht nur positive Effekte für die Effektivität von Online-Lehre und Präsenzlehre, auch vor dem Hintergrund des lebenslangen Lernens in einer sich transformierenden Arbeitswelt sind diese Kompetenzen relevant [4].

Die Maßnahmen sind strukturell durch die Hochschul- und Studiengangsleitung, aber auch durch das Studentische Gesundheitsmanagement zu begleiten und weiterzuentwickeln, damit das Studium nicht als gesundheitliche Belastung wahrgenommen wird.

### Grenzen der Untersuchung

Die hier dargestellten empirischen Zusammenhänge beruhen auf eine Querschnittstudie. Ursache-Wirkungs-Zusammenhänge können anhand der Querschnittstudie nicht empirisch belegt werden. Für die Zukunft wäre es wünschenswert, dass mittels einer Panelstudie die Verlaufsformen von differenzierten psychischen Belastungserfahrungen, verschiedenen Ressourcen und Beanspruchungsfolgen von Studierenden erhoben werden, um noch gezielter empirische Analysen für die Gesundheitsförderung und Prävention durchführen zu können.

Die Identifikation des psychosozialen Mechanismus zwischen Belastungserfahrungen, Ressourcen und Beanspruchungsfolgen in einer Panelstudie kann helfen, gezielt gesundheitsfördernde Maßnahmen zu entwickeln, so dass Studierende in Zukunft bei der Erhaltung und Förderung ihrer Gesundheit besser begleitet und unterstützt werden können.

## Fazit für die Praxis

- Maßnahmen, welche die Senkung der pandemiebedingten Belastungserfahrungen und die Stärkung der Resilienz zum Ziel haben, können die depressive Stimmung von Studierenden senken und damit zu einem höheren Wohlbefinden beitragen. Mögliche Maßnahmen sind folgende:
- regelmäßige Erhebung von psychischen Belastungen und Beanspruchungsfolgen von Studierenden und die Etablierung von entsprechenden Reflexionsformaten,
- Einrichtung und Austausch zwischen Studierenden und Lehrenden auf Studiengangsebene organisieren, um die pandemiebedingten Belastungserfahrungen zu identifizieren und zu mindern,
- Studierende über Resilienz und Wege zur Resilienz informieren, um ein Bewusstsein zu schaffen,
- Resilienzprogramme für Studierende anbieten,
- Ausbau und Verweis auf psychosoziale Angebote der Hochschule, die nicht nur Angebote zum Abbau von Belastungserfahrungen im Hochschulkontext anbietet, sondern auch gezielt Angebote entwickelt, um pandemiebedingte Belastungserfahrungen zu mindern.
